# Management of bronchial secretions with Free Aspire in children with cerebral palsy: impact on clinical outcomes and healthcare resources

**DOI:** 10.1186/s13052-016-0216-0

**Published:** 2016-01-20

**Authors:** Giancarlo Garuti, Elisa Verucchi, Isabella Fanelli, Michele Giovannini, Joao Carlos Winck, Mirco Lusuardi

**Affiliations:** Pneumology Unit, Santa Maria Bianca Hospital, via Fogazzaro 1, Mirandola (MO), 41025 Modena, Italy; Italian Union against Muscular Dystrophy (UILDM), Modena and Reggio Emilia Sections, Modena, Italy; Public Health Care Department, Child Neuropsychiatric Unit, Modena, Italy; Faculdade de Medicina, Universidade do Porto, Porto, Portugal; Respiratory Rehabilitation Unit, S. Sebastiano Hospital, Correggio (RE), and UILDM, Reggio Emilia, Italy

**Keywords:** Cerebral palsy, Bronchial secretions, Chest physiotherapy, Primary healthcare

## Abstract

**Background:**

Management of secretions in children with cerebral palsy is often problematic due to severe deformation of the rib cage, impaired cough, and patients’inability to collaborate with chest physiotherapy. Assessing the effectiveness of different methods and techniques of secretion clearance is hampered by the lack of direct outcome measures and by limited patient cooperation.

This observational study was planned to evaluate the efficacy of Free Aspire, a device that utilizes a special method to remove secretions from the bronchial tree in hypersecretive patients.

**Case presentation:**

Cerebral palsy patients were selected who had experienced more than 3 episodes of respiratory exacerbations in the latest year despite therapeutic optimization (including bronchial clearance techniques) and who had received at least one antibiotic course or underwent at least one access to the Emergency Room (ER) or admission to hospital in the 6 months prior to the study. Patients with congestive heart failure or contraindications for Free Aspire were excluded. We prospectively enrolled 8 patients (mean age 8.25 ± 6.11 years) who had been using in the past techniques for clearance secretions different from Free Aspire.

The treatment with Free Aspire consisted of at least two 20-min sessions per day. The observational study period was 18 months.

In the 6 months prior to start the treatment (T0), patients had a mean number of 4.0 ± 2.23 visits from the primary care pediatrician (PCP), spent 14 ± 20 days in hospital, and received antibiotics for 35 ± 17 days. After the first 6 months of treatment (T1), they had 1.7 ± 0.73 PCP visits, no days spent in hospital, and 9.75 ± 10.4 days of antibiotic therapy. At 12 months of treatment (T2), PCP visits were 1.7 ± 0.70, days in hospital 1.12 ± 0.3, and days of antibiotics 10.25 ± 10. At 18 months of treatment (T3) no hospitalizations had occurred, PCP visits were 0.25 ± 0.70, and days of antibiotic therapy 4.8 ± 12.62. The technique proved to be safe and well tolerated.

**Conclusion:**

Our findings show that Free Aspire for bronchial secretion clearance in cerebral palsy patients with limited capacity to collaborate is safe and effective in reducing the impact of respiratory exacerbations in terms of number of PCP visits, days spent in hospital, and days of antibiotic therapy; its regular use maintains this effect in time.

## Background

Cerebral palsy (CP) is the most frequent cause of motor handicap in children. The prevalence of CP is stable and it is estimated to affect 2 to 3 per 1000 births in Europe [1]. The life expectancy for individuals affected by CP is generally lower that age-matched cohorts of healthy subjects, but varies according to the severity of the motor and cognitive handicap. European data suggest that CP is associated to comorbidities such as severe mental retardation in 31 %, epilepsy in 21 %, severely retarded development with inability to walk in 20 %, and blindness in 11 % of cases [2, 3].

**Fig. 1:**
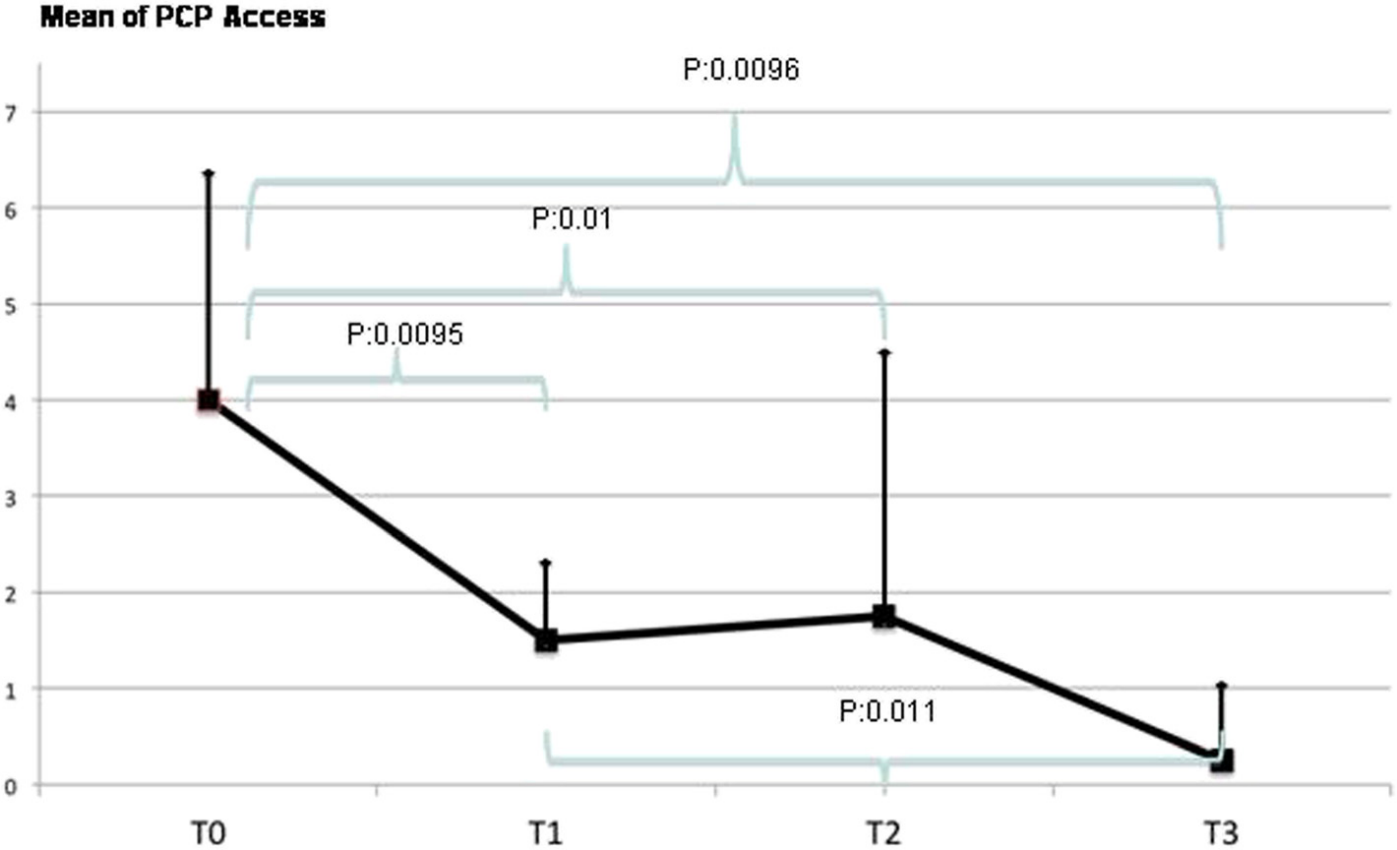
Home visits by the primary care pediatrician (PCP) for respiratory problems

**Fig. 2:**
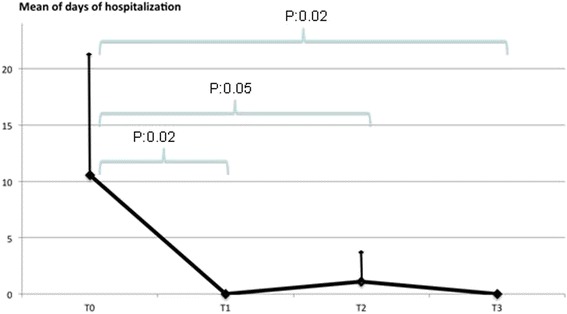
Days spent in hospital for respiratory problems

**Fig. 3:**
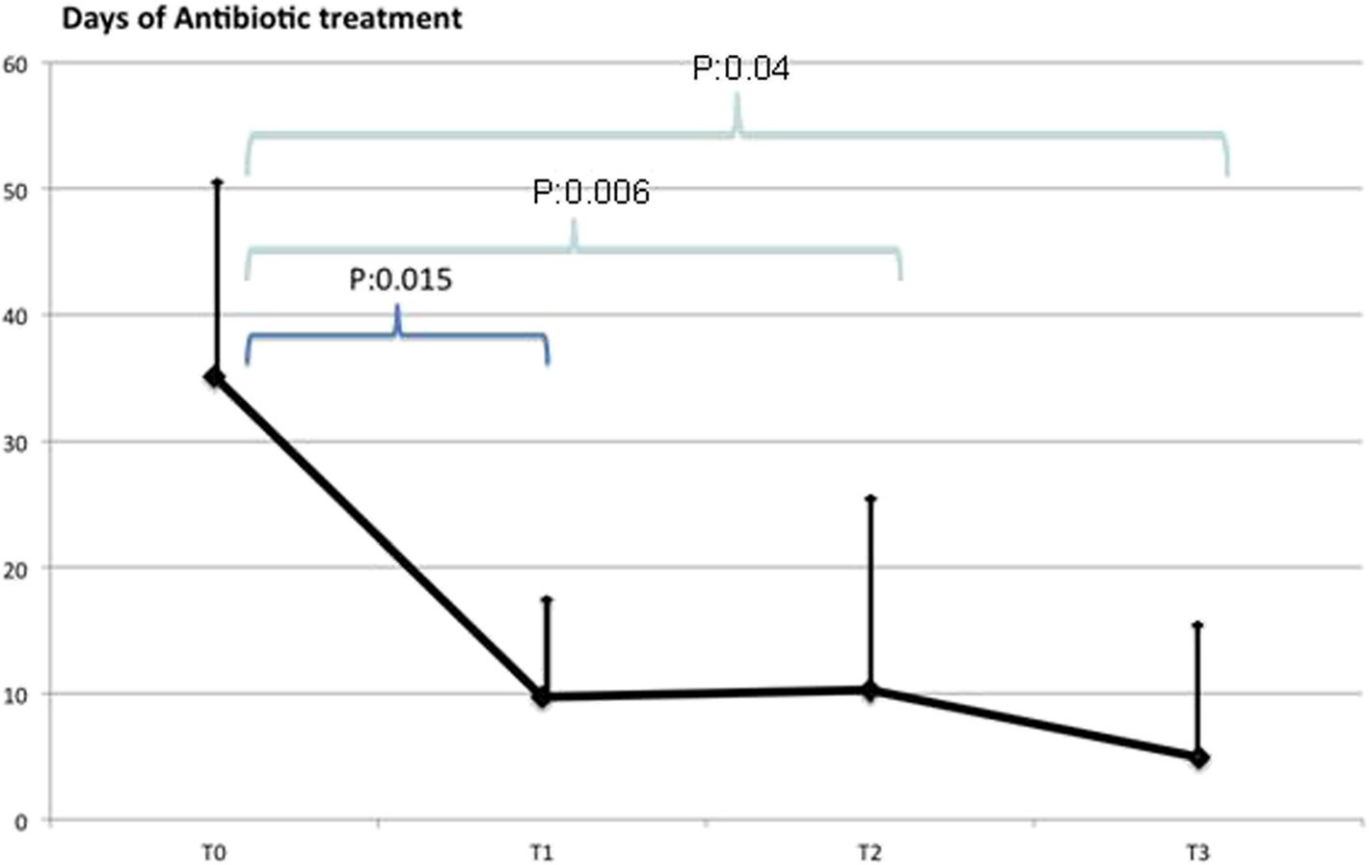
Days of antibiotic treatment

Children with severe neurological impairment have a high incidence of different respiratory disturbances and. respiratory failure is actually one of the most frequent causes of death [4]. Baikie et al. studied 63 spastic tetraplegic children unable to walk, in order to document the prevalence of pulmonary aspiration and the best method for diagnosis, and found that 56 % underwent aspiration causing infection [4, 5]. Reddihough et al. published a survey from the Infantile Cerebral Palsy registry in Victoria, Australia: the majority of 155 children who died between 1970 and 1995 had had severe respiratory problems. Respiratory symptoms were common in these patients, with daily cough or wheezing in 58 %, cough during feeding at least 1 day/week in 84 %, episodes of asthma in the previous 6 months in 34 %, and snoring in 44 % with apnea recorded in 10 %. Respiratory symptoms were present in 24 %, with cough when drinking milk found in 44 %; at the physical examination rales were reported in 19 % and wheezing in 17 % of patients [6].

Depending on the severity of the disorder, ineffective cough and impaired removal of secretions, children with CP can present early in life recurrent infections of the airways, pneumonia, and atelectasis [7]. These can cause airways obstruction and lung damage, and further impair the respiratory mechanics, as well as the nutritional status [8]. Children with CP can develop gas exchange abnormalities (hypercapnia and hypoxemia) and sleep-associated respiratory disturbances, similarly to patients with neuromuscular diseases. Both conditions can develop scoliosis due to the altered muscular tone [7, 8]. The reduced chest wall compliance and asimmetrical lung expansion aggravate the lung impairment. The deformity of the spinal column can further reduce the capacity to cough and the clearance of lung secretions [8]. Hence, children with CP are susceptible to respiratory diseases for several reasons: 1) a tendency to aspire food or liquid on account of swallowing dysfunction, with or without gastroesophageal reflux; 2) a tendency to scoliosis, and rotational deformities of the airways that lead to disventilation of the lower lobes; 3) cough impairment; and 4) a tendency to reduced nasopharyngeal muscle tone which leads to upper airways obstruction and obstructive sleep apnea syndrome.

While several methods exist that allow the management of airways secretions in a comfortable, noninvasive mode, these are nevertheless influenced by the patient’s cognitive status and ability to cooperate [9–11]. An efficient drainage of respiratory secretions through a “vacuum” effect has been found to be effective and safe in patients with chronic obstructive pulmonary disease (COPD) and neuromuscular disorders [12, 13]. The effectiveness of such a treatment in pediatric patients with CP is still under debate [11].

Free Aspire is a device for the non-invasive removal of tracheobronchial secretions, developed for adult and paediatric patients with reduced or no capacity to cough and expectorate.

To determine whether Free Aspire is safe and well tolerated and can facilitate mucociliary clearance and thus, improve respiratory health in the long term, we prospectively studied pediatric patients with CP and severe lung impairment due to retention of bronchial secretions.

## Case presentation

Among subjects followed up at a respiratory outpatient clinic for Rehabilitation of severe infant disabilities, patients with CP aged > 2 years, either male or female, were consecutively enrolled for this study according to the following inclusion criteria:

1. diagnosis of CP by a qualified medical specialist;
2. absence of acute respiratory distress;
3. frequent episodes of respiratory exacerbations (>3/year) despite optimized therapy and bronchial clearance with devices different from Free Aspire
4. at least one antibiotic course or one access to ER or hospital admission in the 6 months preceding the study.

Patients who had congestive heart failure or contraindications for Free Aspire (i.e. neck lesions, incapacity to maintain an upright position, facial deformities, and hemodynamic instability) were excluded from the study.

Written informed consent to the treatment protocol was obtained from the parents or legal tutors of the patients based on the guidelines of the Regional Healthcare System of Emilia Romagna and the local healthcare services of Reggio Emilia. With the start of the study, baseline data were collected, including the history of hospitalizations and antibiotics used for respiratory complications during the previous 6 months (Table 1). The project was approved by institutional review board.

**Table 1:** Demographic and clinical characteristics of the study population (*n* = 8)

Demographics	
Age, years (mean ± SD)	8.25 ± 6.11
Sex, M/F	8/0
Racial/ethnic group	
Caucasian	6
North African	2
Clinical history	N. patients
Hospitalized for pulmonary complications (in previous 6 months)	6
ER admissions (in previous 6 months)	6
Primary care pediatrician home visits (in previous 6 months)	6
Oral antibiotics for respiratory exacerbations (in previous 6 months)	8
Scoliosis	8
Spinal Fusion	1
Previous/current home use of airways clearance techniques	8

### Device and study procedure

Free-Aspire (MPR, Medical Products Research S.r.l, Italy) is an electromedical machine for removing broncho-alveolar secretions. The device utilizes Vakűm technology: during expiration the airflow is accelerated by the Venturi effect inside a special connector. The acceleration is activated only during the expiratory phase, is proportional to the flow of air on spontaneous breathing, and does not require any cooperation. The secretions slide along the layer of liquid lining the bronchial epithelium until they reach the glottis from where they are either expctorated or swallowed. No negative pressure is generated inside the airways, avoiding a possible risk of collapse. Free Aspire does not assist, nor does it cause cough; no respiratory effort is required for the elimination of the secretions. Free Aspire can be used for 20 min without causing respiratory fatigue or discomfort.

The caregivers were asked to carry out the therapy for 20 min at least twice daily, for the duration of the study period. A respiratory therapist instructed caregivers in the procedure and monitored the first treatment session. The participants were trained to perform the chest physiotherapy using an oronasal mask, with supplementary sessions in case of persistent hypersecretion.

The observation period of this study lasted 18 months from the start of treatment (T0) with Free Aspire. Evaluation and data collection were carried out at 6 (T1), 12 (T2) and 18 (T3) months, respectively.

#### Clinical outcomes

The set of clinical outcome measures included the number of pediatrician visits, days spent in hospital and days of antibiotic therapy for a respiratory exacerbation; the occurrence of adverse events in the course of the study were registered too.

Control data for comparison were represented by historical data of the same patients as taken by electronic health files of the 6 months before the start of the study.

### Statistical analysis

Since this was a preliminary observational study, it was not powered in terms of any measure identified as the primary outcome. Results are shown as mean ± standard deviation (SD) unless otherwise specified. The analysis of within-group change from baseline to follow-up visits was carried out for each outcome at the same time. The continuous variables for the outcome measures were analyzed using Student’s paired *t*-test. Values of *p* < 0.05 were considered as significant.

### Patient demographic and clinical characteristics

Eight patients were enrolled, all male, with a mean age of 8.25 ± 6.11 (SD) years.

The primary causes of CP were: prematurity (*n* = 3), cerebral asphyxia (*n* = 2), and meningitis (*n* = 2); in 1 subject the cause was unknown. Patients were under the care of a local Neuropsychiatry unit. Multidisciplinary and multiprofessional assessments were carried out by a team including a rehabilitation therapist, an occupational therapist, a physiatrist and a neuropsychiatrist. The diagnosis of CP was established through specialist evaluations by pediatricians and pediatric neurologists, as documented in our electronic datawarehouse for healthcare data. All 8 patients had a diagnosis of global cognitive retardation and impaired development, with the need for full assistance in daily living activities. All presented with spastic tetraparesis and encephalopathy, and were unable to walk. None had undergone non-invasive ventilation, nor had a device for cough mechanical assistance, but in the previous 6 months they had all used a positive expiratory pressure (PEP) mask. All 8 patients had a history of aspiration, as documented either by a diagnosis of aspiration pneumonia or by an abnormal swallowing test. Four patients were fed orally since the parents refused a gastrostomy. Three had documented gastroesophageal reflux disease and were fed through a gastrostomy tube. One had undergone fundoplication. All were receiving medical treatment for gastroesophageal reflux. All 8 patients had moderate/severe scoliosis (>15° Cobb angle). One had undergone spinal correction.

In the 6 months prior to the study, 6 of the 8 patients had been hospitalized, and 6 had accessed the ER for respiratory disturbances; 6 had required a pediatrician visit in the previous 6 months on account of respiratory symptoms. All subjects had been prescribed oral antibiotics for respiratory complications in the 6 months prior to the start of the study (Table 1).

### Outcome measures

All participants completed the study and were included in the final analysis. No adverse events were registered during the course of the study, such as pain or distress or difficulty in tolerating the therapy.

With respect to baseline a statistically significant reduction, was observed in the home visits of the primary care pediatrician for respiratory exacerbations (Fig. 1): 4.01 ± 2.23 at T0 compared to 1.5 ± 0.92 at T1 (*p* = 0.0095), 1.75 ± 3.05 at T2 (*p* = 0.01), and 0.25 ± 0.70 at T3 (*p* = 0.0096). While the difference between T1 and T3 (*p* = 0.011) was again significant, those between T1 and T2 (*p* = 0.81), T2 and T3 (*p* = 0.22) were not statistically important.

Regarding days spent in hospital (Fig. 2), in the 6 months before the study the mean stay in hospital was 9.81 ± 10.55 days compared to zero days in the 6 months following treatment; at 12 months (T2) the mean stay in hospital was 0.75 ± 1.48 days, to become zero again at 18 months. Comparing T0 with T1 the difference was significant (*p* = 0.02), as were T0 versus (vs) T2 (*p* = 0.05), and T0 vs T3 (*p* = 0.02). The differences T1 vs T2 (*p* = 0.19), T1 vs T3 (*p* = 0.9) and T2 vsT3 (*p* = 0.19) were not significant.

The use of antibiotics (Fig. 3) showed the same trend. From a mean 35.14 ± 17.99 days of antibiotic treatment in the 6 months prior to the study (T0), the use of antibiotics decreased to 9.75 ± 8.31 days in the following 6 months (T1); at T2 days of antibiotic use were 10.25 ± 16.20, and at T3 4.8±12.62. Also in this case, the difference T0 vs T1 was significant (p =0.015), as were the differences T0 vs T2 (*p* = 0.006), and T0 vs T3 (*p* = 0.04). The comparisons T1 vs T2 (*p* = 0.72), T1 vs T3 (*p* = 0.40), and T2 vs T3 (*p* = 0.44) were not statistically significant.

## Discussion

Cerebral palsy (CP) is the most frequent cause of motor handicap observed in childhood and is frequently associated with respiratory problems such as ineffective cough, pulmonary aspiration and sleep-disordered breathing. An altered secretion clearance can lead to the development of recurrent pulmonary infections, bronchiectasis and, eventually, to respiratory failure. While in infantile neuromuscular disorders there are many articles published on the management of similar respiratory problems, including the use of devices for chest physiotherapy, the literature on CP is scant [14, 15]. Methods such as the cough machine, PEP mask and chest vibrations are now common in neuromuscular patients [16–18]. Among few studies on chest physiotherapy in CP [19], Plioplys et al. in 2002 [20] investigated the routine use of high-frequency chest wall compression (using the Vest system) in a prospective non-controlled study in 7 children with CP (5 of whom had a tracheostomy) resident in a specialized pediatric care facility. The total number of pneumonia episodes and antibiotic courses diminished from 36 per year before Vest to 18 during the year of treatment, and the number of pneumonia-related hospitalizations decreased from 9 to 3. Another study published by Yuan et al. [21] shows that also high-frequency external vibrations were able to reduce the number of hospital admissions in patients with CP and neuromuscular diseases compared to controls. A recent Cochrane review analyzed the efficacy of non-pharmacological treatments for respiratory problems in children with severe retardation of global development: 15 studies of relevance were identified. The findings, even if related to heterogeneous treatments, showed that there were potential benefits, and for most of the interventions no severe adverse events were reported. The conclusion was that, irrespective of the type of treatment, devices for the removal of secretions in severe cerebral disability are potentially useful [22].

To date, no studies have been published evaluating the use of Free Aspire in infantile CP or generally in pediatric patients and, to our knowledge, this is the first observational study on this particular patient group treated with this device. The advantage of using the Vakűm method is that the patient does not have to perform coordinated and stressful respiratory maneuvers. The results of the study are extremely positive in terms of major outcomes such as home visits of the primary care pediatrician, days of hospitalization and use of antibiotics for respiratory exacerbations. The improvement was particularly evident in the first 6 months of observation, and persisted in the following 6 to 12 months. In the 18 months of clinical observation no patient abandoned the treatment due to adverse events, such as pain or distress or difficulty in tolerating the therapy, therefore demonstrating that the technique is well tolerated and safe.

A limitation of our study is the small patient sample: despite our center represents a referral unit for pediatric chest physiotherapy, we were able to recruit only a group of 8 patients with cerebral palsy, all of whom, however, completed the period of observation. A further weakness is represented by the longitudinal observational design and comparison with historical data of the same patients, instead of a parallel group randomized trial that would have required a larger number of subjects. Anyway, patients had been on follow up at the same center with the same health professionals and the same approach for the two periods on comparison, therefore the only variable was just the different instrumentation used for secretion removal.

## Conclusion

From our findings, it can be concluded that the application of Free Aspire to improve the bronchial secretion clearance in non-collaborating patients with CP, is feasible and effective in reducing respiratory complications and exacerbations, as well as the utilization of healthcare resources (PCP visits, days of hospitalization, need for antibiotics). Importantly, the regular use of the device maintains these results in time. Further studies are necessary on how to improve the mucociliary clearance and thus optimize the respiratory condition of children with CP; to this end, randomized controlled studies in larger patient cohorts are required.

## Abbreviations

CP: Cerebral palsy
ER: Emergency room
PCP: Primary care pediatrician
NIV: Non-invasive ventilation
PEP: Positive expiratory Pressure
